# Semen quality from patients affected by seminomatous and non-seminomatous testicular tumor

**DOI:** 10.1590/S1677-5538.IBJU.2021.99.01

**Published:** 2020-05-10

**Authors:** Rosana Xavier, Renata Cristina de Carvalho, Renato Fraietta

**Affiliations:** 1 Universidade Federal de São Paulo Divisão de Urologia Departamento de Cirurgia São PauloSP Brasil Departamento de Cirurgia, Divisão de Urologia, Universidade Federal de São Paulo - UNIFESP São Paulo, SP, Brasil.

**Keywords:** Testicular Neoplasms, Infertility, Male, Seminoma

## Abstract

Testicular cancer is considered a rare disease affecting approximately 1% to 2% of the male population. This neoplasm has a cure rate of over 95%; as a result, a major concern is the future of fertility of carriers from this disease. There are several histological subtypes of testicular tumors; however, the Testicular Germ Cell Tumors (TGCTs), comprising both seminoma and non-seminoma tumors, are considered the main subtypes of testicular neoplasms. TGCT are characterized by being a solid tumor that mostly affects young men aged between 15 and 40 years old. While TGCT subtypes may have an invasive potential, seminoma subtype does not affect other cells rather than germ cells, while non-seminomas have more invasive properties and can achieve somatic cells; thus, having a more aggressive nature. This research intends to review the literature regarding information about sperm parameters, correlating the data found in those studies to the subfertility and infertility of patients with TCGTs. Furthermore, it will also correlate the data to the non-seminoma and seminoma histological subtypes from pre- and post-cancer therapy. PubMed databases were used. Searched keywords included: seminoma AND non-seminoma; male infertility; germ cell tumor; chemotherapy AND radiotherapy. Only articles published in English were considered. Current studies demonstrate that both TGCT subtypes promote deleterious effects on semen quality resulting in decreased sperm concentration, declined sperm total motility and an increase in the morphology alterations. However, findings suggest that the non-seminoma subtype effects are more pronounced and deleterious. More studies will be necessary to clarify the behavior of seminoma and non-seminoma tumors implicating the reproductive health of male patients.

## INTRODUCTION

Testicular cancer is considered a rare disease that affects approximately 1% to 2% of the male population (1). This neoplasm has a cure rate of over 95%, as a result, a major concern is the future of fertility of carriers from this disease. Furthermore, there is a growing body of evidence that testicular tumor and infertility are deeply related, with infertile men having a higher chance of also presenting testicular cancer (2, 3).

**Figure 1:**
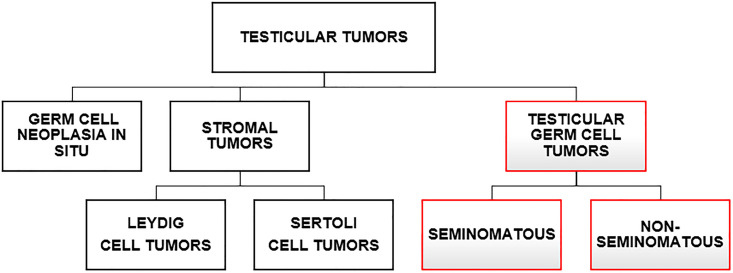
Testicular tumor types. The types of cancer that can affect the testicles are in the figure. We can divide them into two major groups: stromal tumors (Leydig cells and Sertoli cell tumors) and germ cell tumors having the germ cell neoplasia in situ (GCNIS) as a common precursor.

There are several histological subtypes of tumors which may affect the testis including: germ cell neoplasia in situ (GCNIS), previously known as carcinoma *in situ*, stromal tumors (Leydig cell and Sertoli cell tumors), and testicular germ cell tumors (TGCTs), with seminoma and non-seminoma tumors (Figure-1) considered the main subtypes of testicular neoplasms (3). Importantly, both are derived from a common precursor, the in situ germ cell neoplasia of the testis (4, 5).

TGCT is characterized by a solid tumor that mostly affects young men aged between 15 and 40 years old. The worldwide incidence of this disease is 1.5 cases per 100.000 men; however, it can differ between countries and breeds, presenting a higher incidence in Scandinavian countries, Switzerland and Germany; yet, in African countries, Asians and Latin populations, it has demonstrated a very low incidence (3, 6, 7).

Although the exact cause of its occurrence is not yet defined, some elements are known as associated risk factors, which are: cryptorchidism (3), infertility, contralateral tumor presence and genetic factors (8). Among the genetic causes, it has already been described that 80% of the TGCTs are featured by an addition in the short arm of 12p chromosome as a isochromosome i (9). Thus, the insertion in the 12p chromosome leads to a relevant functional alteration, promoting the stem cell maintenance and proliferation by the key genes activations, as: POU5F1/OCT3/4, SOX2 e NANOG (10). Mutations in the SRY gene are connected to the gonadal tumor development, gonadal dysgenesis and infertility, which may occur in approximately 52.5% of the individuals presenting an alteration in this gene (11). Another studied gene is the tumor suppressor p53, which has an important role in spermatogenesis; it is know that p53 mutations leads to a genomic and chromosomal instability that can compromise sperm fecundity ability in the oocyte; further, it is related to TCGTs pathogenesis (12). Studies have shown that genetic factors along with epigenetics plays an important role in the neoplasm development (8, 13). The epigenetics regulatory processes occurs in the beginning and protection pluripotency mechanisms of embryonic stem cells and also in identity maintenance of differentiated cell types (14).

Skakkebaek in 1993 hypothesized that an increase in anomalies incidence of male reproductive function could be related to increased estrogen exposure in utero. Highlights the diethylstilbestrol (DES), a powerful synthetic estrogen indicated to pregnant women with repeat abortion history, being associated with increased risk of hypospadia, cryptorchidism and low quality sperm in the offspring (15). Chemical and xenoestrogens products that promote endocrine dysregulation may constitute a mediation factor that connects the testicular cancer and infertility; these agents are antiandrogenic and cause an increased in estrogen levels inhibiting the hypothalamus-pituitary-gonadal axis (HPG) resulting in decreased Follicle Stimulating hormone (FSH) production and affecting the amount of Sertoli Cells between the fetal phase and pre pubertal phase (12).

The TGCTs initial diagnosis is performed by a doctor through physical examination, ultrasound and analysis of serum tumor marker increase, such as: alpha fetoprotein (AFP), human chorionic gonadotropin (hCG) and lactic dehydrogenase enzyme (LDH) (16, 17). The AFP is a glycoprotein with 70kDa produced by fetal yolk sac, liver and gastrointestinal tract. Higher levels of this glycoprotein are usually found in non-seminoma tumors. When hCG levels are increased, it may indicate the presence of seminoma and non-seminoma tumors and of the above markers, the LDH is the least specific; however, evidence of its association with the tumor has increased (18).

The availability of effectives therapies and the discovery of sensitive tumor markers help in disease treatment, raising the healing chances up to 97%. The treatment for TGCTs consists of: radical orchiectomy, resection of retroperitoneal lymph nodes, chemotherapy and radiotherapy (3, 19).

**Figure 2:**
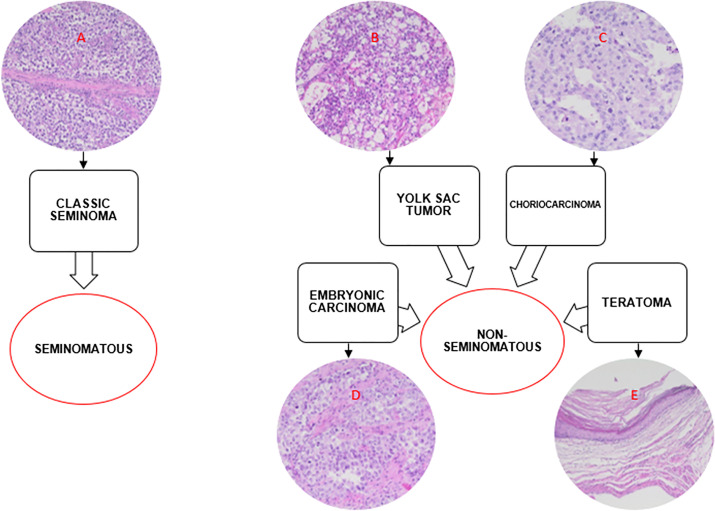
Seminoma and non-seminoma tumors subtypes and their histological representations. The histological representation was prepared by hematoxylin and eosin (HE) method. The analysis was done under 200x magnification and the images were provided by the Department of Pathology of the São Paulo Federal University. A) Presence of inflammatory infiltrate, consisting of uniform cells, divided into thin trabecular septa. B) Microcystic pattern; cells compressed by the presence of vacuoles. C) Presence of syncytio-trophoblast cells with multiple mitoses. D) Presence of neoplastic cells forming a solid array. E) Mature teratoma with predominance of keratinized squamous epithelium; presence of epidermoid cyst containing keratin in the lumen.

The subtypes of TGCT may have an invasive potential but seminoma type does not surpass spermatogenic cells. On the other hand, non-seminomas, apart from invading seminoma germ cells, are capable of targeting somatic cells, thereby presenting a more aggressive nature (8). Embryonic carcinoma, yolk sac tumor, choriocarcinoma and teratoma are part of the non-seminoma group (Figure-2) (20).

It has been reported that semen quality of patients with TGCTs is affected, especially in cases of non-seminoma tumors; however, little is known about the mechanisms leading to this alteration (21, 22). For several reasons, subfertility is common among TGCTs patients since 50% of them have abnormal sperm counts (23), which also emphasizes that chemotherapy and radiotherapy treatments have a negative impact on fertility (24).

Around 240 reviews were published with this topic that are extremely useful to clinical research; so, the authors are advancing gradually in the different aspects and contributing to new findings about this neoplasm. Nevertheless, it is important to investigate the differences between the histological subtypes seminoma and non-seminoma since how each one affects seminal quality is not understood yet. Therefore, this research intends to review the literature regarding information about sperm parameters, correlating the data found in those studies to the subfertility and infertility of patients with TCGTs. Furthermore, it will also correlate the data to the non-seminoma and seminoma histological subtypes from pre- and post-cancer therapy.

## MATERIAL AND METHODS

To construct this narrative review, we have used the PubMed database. The inclusion criteria were: papers that evaluated spermatic parameters of seminoma and non-seminoma tumors patients and which made a comparative analysis between the two histological subtypes, pre and post orchiectomy and adjuvant treatment; the exclusion criteria were: papers that did not analyze the seminal parameters and which studied non-germ tumors of the testis. The keywords searched for included: seminoma; non-seminoma; male infertility; germ cell tumor and testicular neoplasms. Only papers published in English were considered.

As a search strategy we have selected four research terms that were combined with the boolean descriptor “AND”, for example: “seminoma AND non-seminoma; testicular neoplasms AND infertility and sperm quality AND germ cell tumor”. We started the searches in February 2019 and we did not stipulate a publication period of time due to the scarcity of relevant articles according to our inclusion criteria; also, we did not find meta-analyzes with the comparison between the histological subtypes; thus, just retrospectives studies, and prospective and systematics reviews were selected.

## RESULTS

After putting into practice the search strategy using the terms combination, we have found 1511 related papers and through the abstracts we have selected 44 papers that were considered relevant and out of these, only 5 articles match the inclusion and exclusion criteria as described above.

### TGCTs and fertility potential

Spermatogenesis is the process through which sperm is produced, so any interference in this process can impair cell division and maturation, affecting or not male fertility potential (25). TGCTs arise from a failure in the maturation of gonocytes, which are primary embryonic cells that give rise to germ cells; thus, it is essential to understand the mechanisms related to spermatogenesis to broaden information on the emergence and distinctions of this malignancy (26).

It is believed that testicular tumors promote a negative effect on the spermatogenesis due to disruptions in the blood-testicular barrier, followed by antibodies formation against the sperm and long lymphocytic infiltrates into the parenchyma adjacent to tumor germ cells; it is known that antisperm antibodies are associated with count decrease, low motility and morphological anomalies of the sperm (27).

It is essential to consider the fertile potential of these patients, since the occurrence of testicular cancer coincides in most cases with the male reproductive phase. Adjuvant treatment is often used, and some patients will become azoospermic after chemotherapy or radiotherapy (24). However, it was also reported that even before treatment, some patients already had poor semen quality, which leads us to believe that the presence of the tumor interferes with spermatogenesis. Indeed, half of the patients in this study revealed oligozoospermia or azoospermia, as described by Ping et al. (28). A possible explanation to spermatogenesis decline in the ipsilateral and contralateral testicle, before the tumor treatment, is not well established. Some causes indicate the inclusion of paracrine and autocrine factors produced by the tumor, cryptorchidism or antispermatic antibodies (29). Besides that, several studies have already demonstrated that the semen quality of the patients with TCGT tends to be damaged in relation to men considered normozoospermic by the WHO (2010) (30).

The adjuvant treatment utilization is common; thus, a high rate of patients will become azoospermic (10 to 24%) (31). Andrade et al. (2019) investigated the semen quality of patients before and after orchiectomy, and in both groups, there was a decrease in ejaculated volume (54.2%) and sperm concentration (50.0%), motility (50.0%) and morphology (20.8%), but it is necessary to consider the low sample size used (total of 24 men: 15 with seminoma tumor and 9 with non-seminoma tumor) (32).

It is important to note that most carriers of this disease are not aware that adjuvant treatment for testicular cancer may lead to persistent azoospermia. Therefore, it is recommended that these patients receive a correct medical advice in order to preserve their fertility for a future parenthood (31). Thus, patients who wants to attain parenthood should be advised to adhere to semen cryopreservation for its later use in assisted reproduction techniques (32).

One study showed that when the tumor affects more than 50% of the testis, the probability of these patients to present a normal spermatogenesis in the affected testis is less than 50%. However, when sperm production occurs, it is located far from the neoplasia (33). Despite the relevance of these tumors in human reproduction, no studies were performed to analyze testicular tumor effects on semen quality, considering the seminomatous and non-seminomatous subtypes separately.

### Seminoma tumor

Considered a malignant germ cell tumor, developed from primordial germ cells (gonocytes), this neoplasia corresponds to about 50% of all TGCTs (3). It tends to grow and spread slowly when compared to the non-seminoma subtype (34). This tumor subtype affects the HPG; thus, disturbing spermatogenesis and promoting and worsening of the patient’s fertile potential (35).

Dias et al. (2019) evaluated the semen of seminoma tumor patients and control individuals (fertile volunteers); 15 samples per group were included. To seminoma group, the analysis was performed before the adjuvant treatment. Their results for semen volume were nearly identical between groups. On the other hand, a decrease in motility (P=0.019), sperm concentration (P=0.003), total sperm count (P=0.001), and total motility (P=0.001) was observed in patients with seminoma. However, both groups are still considered normozoospermic, according to WHO 2010 (36).

Sposito et al. (2017) evaluated semen of 52 patients, 12 with seminoma tumor, 12 with non-seminoma tumor and 26 belonging the control group (healthy men) and did not find significant differences in the parameters of sperm motility and volume. However, sperm concentration, morphology and total count showed a significant decrease compared to the control group (37).

### Non-seminoma Tumor

This tumor subtype presents characteristics of embryonic and extraembryonic differentiation. It has had a worse prognosis and a higher metastatic potential in the short term compared to the seminoma subtype (38). Approximately half of the cases of TGCT are diagnosed as this subtype (39).

Fraietta et al. (2010) analyzed semen samples of 100 patients, out of these 37 samples had been detected with seminoma tumor and 63 with non-seminoma tumor, and it was observed that the values for total motile morphologically normal sperm were lower (P=0.022) in non-seminoma patients. Accordingly, Liguori et al. (2008) evaluated the semen quality of 30 patients in the moment pre and post orchiectomy, and found that sperm concentration was significantly lower in patients with non-seminoma tumor (17.9 x 106/ml and 8.16 x 106/ml) compared to the seminoma subtype (35.47 x 106/ml and 23.99 x 106/ml) (P=0.004). Therefore, non-seminoma tumors have had a negative effect on semen quality which is more pronounced than in seminoma tumors (21, 22).

Dias et al. (2019) compared semen samples of 15 healthy men with 15 cryopreserved samples from patients with non-seminoma tumors prior to cancer therapy and the results were: volume and motility were similar between groups; on the other hand, sperm concentration and total count decreased significantly compared to the control group (P=0.0015 and P=0.0084, respectively). These findings confirm the hypothesis of sperm quality deterioration in this group of patients (40).

Table-1 shows studies that executed the semen parameters comparison between the tumor subtypes of seminoma and non-seminoma.

**Table 1:** Studies with seminal parameters analysis according the histological tumor types seminoma and nonseminoma.

Study group			Parameters									
Authors	Histological tumor subtypes	Comparator	Semen volume (ml)	Sperm motility (%)	Total motile count (10^6^/mL)	Non-progressive motility (%)	Total motile morphologically normal sperm (10^6^/mL)	Sperm concentration (10^6^/mL)	Total sperm count (10^6^/mL)	Morphology (% normal)	Round cells 10^6^/mL	Neutrophils 10^6^/mL
Dias et. al (2019) (35)	Seminoma	Control	p= 0.541	p= 0,019 ↓	p= 0.001 ↓	X	X	p= 0,003 ↓	p= 0.001 ↓	X	X	X
Sposito et. al (2017) (36)	Seminoma/Nonseminoma	Control	p= 0.478	p= 0.098	X	p= 0.340	X	p= <0.0001 ↓	p= <0.0001↓	p= <0.0001↓	p= 0.304	p= 0.577
Fraietta et. al (2010) (20)	Nonseminoma	Seminoma	p= 0.0943	p= 0.0644	X	X	p= 0.022 ↑	p= 0.576	X	p= 0.105	p= 0.868	p= 0.822
Liguori et. al (2008) (21)	Nonseminoma	Seminoma	X	X	X	X	X	p= 0.004 ↓	p= 0.001↓	X	X	X
Dias et. al (2019) (39)	Nonseminoma	Control	p= 0.7044	p= 0.6936	p= 0.0136↓	X	X	p= 0.0015 ↓	p= 0.0084↓	X	X	X

Statistically significant decrease; ↑ Statistically significant increase; X Parameter not analyzed.

### Treatment for TGCTs

There are evidences from the literature that after unilateral orchiectomy, sperm concentration becomes reduced (average sperm concentration: 16.6 x 106/mL), compared to the results from the pre-surgery time (average sperm concentration: 26.7 x 106/mL) (22, 29); at the same time, the resection of retroperitoneal lymph nodes promotes a dry ejaculation in virtually 100% of the patients (41). Radiation therapy and chemotherapy are considered cytotoxic and can have a significant effect on the fertile potential. However, radiotherapy has a higher eradication rate, while chemotherapy varies according to dose and cycles (42). Nevertheless, the latter is considered the least deleterious to fertility.

Approximately 50% of testicular tumor patients tend to have an impaired semen quality at the onset of the disease (23). However, after the adjuvant treatment for cancer, the condition can aggravate and 30% of patients present a negative effect on fertility (43).

## CONCLUSIONS

More studies are necessary to clarify the behavior of seminoma and non-seminoma tumors implicating the reproductive health of male patients. Current findings show that both subtypes promote deleterious effects on the sperm parameters and some reports show that the non-seminoma subtype may lead to more impaired semen quality. Therefore, a better understanding of the molecular and pathophysiological mechanisms of TGCTs is necessary to enable the distinct measurement of how these subtypes interfere with the patient’s fertility potential.

## ABBREVIATIONS

TGCT: Testicular Germ Cell Tumor
DES: Diethylstilbestrol
HPG: Hypothalamus-pituitary-gonadal axis
FSH: Follicle Stimulating hormone
AFP: Alpha fetoprotein
hCG: Human chorionic gonadotropin
LDH: Lactic dehydrogenase
WHO: World Health Organization
GCNIS: Germ cell neoplasia in situ

## References

[1] 1. Siegel RL, Miller KD, Jemal A. Cancer statistics, 2018. CA Cancer J Clin. 2018;68:7-30. 10.3322/caac.21442 29313949

[2] 2. Sabanegh ES Jr, Ragheb AM. Male fertility after cancer. Urology. 2009;73:225-31. 10.1016/j.urology.2008.08.474 19036419

[3] 3. Moch H, Cubilla AL, Humphrey PA, Reuter VE, Ulbright TM. The 2016 WHO Classification of Tumours of the Urinary System and Male Genital Organs-Part A: Renal, Penile, and Testicular Tumours. Eur Urol. 2016;70:93-105. 10.1016/j.eururo.2016.02.029 26935559

[4] 4. Skakkebaek NE. Possible carcinoma-in-situ of the testis. Lancet. 1972;2:516-7. 10.1016/s0140-6736(72)91909-5 4115573

[5] 5. Kristensen DG, Nielsen JE, Jørgensen A, Skakkebæk NE, Rajpert-De Meyts E, Almstrup K. Evidence that active demethylation mechanisms maintain the genome of carcinoma in situ cells hypomethylated in the adult testis. Br J Cancer. 2014;110:668-78. 10.1038/bjc.2013.727 PMC3915112 24292451

[6] 6. Albers P, Albrecht W, Algaba F, Bokemeyer C, Cohn-Cedermark G, Fizazi K, et al. Guidelines on Testicular Cancer: 2015 Update. Eur Urol. 2015;68:1054-68. 10.1016/j.eururo.2015.07.044 26297604

[7] 7. Chia VM, Quraishi SM, Devesa SS, Purdue MP, Cook MB, McGlynn KA. International trends in the incidence of testicular cancer, 1973-2002. Cancer Epidemiol Biomarkers Prev. 2010;19:1151-9. 10.1158/1055-9965.EPI-10-0031 PMC2867073 20447912

[8] 8. Rajpert-De Meyts E, McGlynn KA, Okamoto K, Jewett MA, Bokemeyer C. Testicular germ cell tumours. Lancet. 2016;387:1762-74. 10.1016/S0140-6736(15)00991-5 26651223

[9] 9. Atkin NB, Baker MC. Specific chromosome change, i(12p), in testicular tumours? Lancet. 1982;2:1349. 10.1016/s0140-6736(82)91557-4 6128640

[10] 10. Korkola JE, Houldsworth J, Chadalavada RS, Olshen AB, Dobrzynski D, Reuter VE, et al. Down-regulation of stem cell genes, including those in a 200-kb gene cluster at 12p13.31, is associated with in vivo differentiation of human male germ cell tumors. Cancer Res. 2006;66:820-7. 10.1158/0008-5472.CAN-05-2445 16424014

[11] 11. Uehara S, Hashiyada M, Sato K, Nata M, Funato T, Okamura K. Complete XY gonadal dysgenesis and aspects of the SRYgenotype and gonadal tumor formation. J Hum Genet. 2002;47:279-84. 10.1007/s100380200040 12111377

[12] 12. Burns WR, Sabanegh E, Dada R, Rein B, Agarwal A. Is male infertility a forerunner to cancer? Int Braz J Urol. 2010;36:527-36. 10.1590/s1677-55382010000500002 21044369

[13] 13. Facchini G, Rossetti S, Cavaliere C, D’Aniello C, Di Franco R, Iovane G, et al. Exploring the molecular aspects associated with testicular germ cell tumors: a review. Oncotarget. 2017;9:1365-79. 10.18632/oncotarget.22373 PMC5787445 29416701

[14] 14. Boland MJ, Nazor KL, Loring JF. Epigenetic regulation of pluripotency and differentiation. Circ Res. 2014;115:311-24. 10.1161/CIRCRESAHA.115.301517 PMC4229506 24989490

[15] 15. Sharpe RM, Skakkebaek NE. Are oestrogens involved in falling sperm counts and disorders of the male reproductive tract? Lancet. 1993;341:1392-5. 10.1016/0140-6736(93)90953-e 8098802

[16] 16. Smith ZL, Werntz RP, Eggener SE. Testicular Cancer: Epidemiology, Diagnosis, and Management. Med Clin North Am. 2018;102:251-64. 10.1016/j.mcna.2017.10.003 29406056

[17] 17. Barlow LJ, Badalato GM, McKiernan JM. Serum tumor markers in the evaluation of male germ cell tumors. Nat Rev Urol. 2010;7:610-7. 10.1038/nrurol.2010.166 21068762

[18] 18. Gori S, Porrozzi S, Roila F, Gatta G, De Giorgi U, Marangolo M. Germ cell tumours of the testis. Crit Rev Oncol Hematol. 2005;53:141-64. 10.1016/j.critrevonc.2004.05.006 15661565

[19] 19. Vaz RM, Bordenali G, Bibancos M. Testicular Cancer-Surgical Treatment. Front Endocrinol (Lausanne). 2019;10:308. 10.3389/fendo.2019.00308 PMC6529845 31156556

[20] 20. [No Authors]. American Cancer Society: What Is Testicular Cancer? | Types of Testicular Cancer. Available at. <https://www.cancer.org/cancer/testicular-cancer/about/what-is-testicular-cancer.html>.

[21] 21. Fraietta R, Spaine DM, Bertolla RP, Ortiz V, Cedenho AP. Individual and seminal characteristics of patients with testicular germ cell tumors. Fertil Steril. 2010; 94:2107-12. 10.1016/j.fertnstert.2009.12.021 20117767

[22] 22. Liguori G, Trombetta C, Bucci S, Benvenuto S, Amodeo A, Ocello G, et al. Semen quality before and after orchiectomy in men with testicular cancer. Arch Ital Urol Androl. 2008;80:99-102. 19009865

[23] 23. Williams DH 4th, Karpman E, Sander JC, Spiess PE, Pisters LL, Lipshultz LI. Pretreatment semen parameters in men with cancer. J Urol. 2009;181:736-40. 10.1016/j.juro.2008.10.023 19091343

[24] 24. Bujan L, Walschaerts M, Moinard N, Hennebicq S, Saias J, Brugnon F, et al. Impact of chemotherapy and radiotherapy for testicular germ cell tumors on spermatogenesis and sperm DNA: a multicenter prospective study from the CECOS network. Fertil Steril. 2013;100:673-80. 10.1016/j.fertnstert.2013.05.018 23755953

[25] 25. Neto FT, Bach PV, Najari BB, Li PS, Goldstein M. Spermatogenesis in humans and its affecting factors. Semin Cell Dev Biol. 2016;59:10-26. 10.1016/j.semcdb.2016.04.009 27143445

[26] 26. Chen SR, Zheng QS, Zhang Y, Gao F, Liu YX. Disruption of genital ridge development causes aberrant primordial germ cell proliferation but does not affect their directional migration. BMC Biol. 2013;11:22. 10.1186/1741-7007-11-22 PMC3652777 23497137

[27] 27. Foster RS, Rubin LR, McNulty A, Bihrle R, Donohue JP. Detection of antisperm-antibodies in patients with primary testicular cancer. Int J Androl. 1991;14:179-85. 10.1111/j.1365-2605.1991.tb01080.x 2066164

[28] 28. Ping P, Gu BH, Li P, Huang YR, Li Z. Fertility outcome of patients with testicular tumor: before and after treatment. Asian J Androl. 2014;16:107-11. 10.4103/1008-682X.122194 PMC3901866 24369141

[29] 29. Petersen PM, Skakkebaek NE, Rørth M, Giwercman A. Semen quality and reproductive hormones before and after orchiectomy in men with testicular cancer. J Urol. 1999;161:822-6. 10022693

[30] 30. De Jonge C. Semen analysis: looking for an upgrade in class. Fertil Steril. 2012;97:260-6. 10.1016/j.fertnstert.2011.12.045 22289285

[31] 31. Lass A, Akagbosu F, Brinsden P. Sperm banking and assisted reproduction treatment for couples following cancer treatment of the male partner. Hum Reprod Update. 2001;7:370-7. 10.1093/humupd/7.4.370 11476349

[32] 32. Andrade MBR, Bertolla RP, Intasqui P, Antoniassi MP, Tibaldi DS, Belardin LB, et al. Effect of orchiectomy on sperm functional aspects and semen oxidative stress in men with testicular tumours. Andrologia. 2019;51:e13205. 10.1111/and.13205 30488474

[33] 33. Kopeika J, Thornhill A, Khalaf Y. The effect of cryopreservation on the genome of gametes and embryos: principles of cryobiology and critical appraisal of the evidence. Hum Reprod Update. 2015;21:209-27. 10.1093/humupd/dmu063 25519143

[34] 34. Moody JA, Ahmed K, Horsfield C, Pedersen MRV, Yap T, Shabbir M. Fertility preservation in testicular cancer - predictors of spermatogenesis. BJU Int. 2018;122:236-42. 10.1111/bju.14214 29667332

[35] 35. Ruf CG, Isbarn H, Wagner W, Fisch M, Matthies C, Dieckmann KP. Changes in epidemiologic features of testicular germ cell cancer: age at diagnosis and relative frequency of seminoma are constantly and significantly increasing. Urol Oncol. 2014;32:33.e1-6. 10.1016/j.urolonc.2012.12.002 23395239

[36] 36. Morrish DW, Venner PM, Siy O, Barron G, Bhardwaj D, Outhet D. Mechanisms of endocrine dysfunction in patients with testicular cancer. J Natl Cancer Inst. 1990;82:412-8. 10.1093/jnci/82.5.412 2304089

[37] 37. Sposito C, Camargo M, Tibaldi DS, Barradas V, Cedenho AP, Nichi M, et al. Antioxidant enzyme profile and lipid peroxidation products in semen samples of testicular germ cell tumor patients submitted to orchiectomy. Int Braz J Urol. 2017;43:644-651. 10.1590/S1677-5538.IBJU.2016.0323 PMC5557439 28266817

[38] 38. Dias TR, Agarwal A, Pushparaj PN, Ahmad G, Sharma R. Reduced semen quality in patients with testicular cancer seminoma is associated with alterations in the expression of sperm proteins. Asian J Androl. 2020;22:88-93. 10.4103/aja.aja_17_19 PMC6958970 31006710

[39] 39. Winter C, Albers P. Testicular germ cell tumors: pathogenesis, diagnosis and treatment. Nat Rev Endocrinol. 2011;7:43-53. 10.1038/nrendo.2010.196 21116298

[40] 40. Oldenburg J, Fosså SD, Nuver J, Heidenreich A, Schmoll HJ, Bokemeyer C, et al. Testicular seminoma and non-seminoma: ESMO Clinical Practice Guidelines for diagnosis, treatment and follow-up. Ann Oncol. 2013;24 Suppl 6:vi125-32. 10.1093/annonc/mdt304 24078656

[41] 41. Dias TR, Agarwal A, Pushparaj PN, Ahmad G, Sharma R. New Insights on the Mechanisms Affecting Fertility in Men with Non-Seminoma Testicular Cancer before Cancer Therapy. World J Mens Health. 2020;38:198-207. 10.5534/wjmh.180099 PMC7076305 30588784

[42] 42. Klein EA. Open technique for nerve-sparing retroperitoneal lymphadenectomy. Urology. 2000;55:132-5. 10.1016/s0090-4295(99)00378-7 10654910

[43] 43. Ruf CG, Gnoss A, Hartmann M, Matthies C, Anheuser P, Loy V, et al. Contralateral biopsies in patients with testicular germ cell tumours: patterns of care in Germany and recent data regarding prevalence and treatment of testicular intra-epithelial neoplasia. Andrology. 2015;3:92-8. 10.1111/j.2047-2927.2014.00260.x 25146646

